# Health Risk for Non-Dietary Children’s Exposure to Heavy Metals in Postindustrial Areas in Upper Silesia, Poland

**DOI:** 10.3390/toxics13050377

**Published:** 2025-05-06

**Authors:** Grzegorz Dziubanek, Joanna Furman, Danuta Rogala, Klaudia Gut-Pietrasz, Małgorzata Ćwieląg-Drabek, Monika Rusin, Joanna Domagalska, Agata Piekut, Renata Baranowska, Anna Niesler, Weronika Osmala-Kurpiewska

**Affiliations:** 1 Department of Environmental Health Risk Factors, Faculty of Public Health in Bytom, Medical University of Silesia in Katowice, 18 Piekarska Street, 41-902 Bytom, Poland; jfurman@sum.edu.pl (J.F.); mdrabek@sum.edu.pl (M.Ć.-D.); mrusin@sum.edu.pl (M.R.); jdomagalska@sum.edu.pl (J.D.); 2 Analytical Laboratory of Department of Environmental Health, Faculty of Public Health in Bytom, Medical University of Silesia in Katowice, 18 Piekarska Street, 42-902 Bytom, Poland; drogala@sum.edu.pl (D.R.); kgut@sum.edu.pl (K.G.-P.); rbaranowska@sum.edu.pl (R.B.); 3 Department of Environmental Health, Faculty of Public Health in Bytom, Medical University of Silesia in Katowice, 18 Piekarska Street, 42-902 Bytom, Poland; apiekut@sum.edu.pl (A.P.); anna.niesler@sum.edu.pl (A.N.); wosmala@sum.edu.pl (W.O.-K.)

**Keywords:** children, soil, non-dietary exposure, heavy metals, postindustrial area, health risks, Upper Silesia, Poland

## Abstract

Heavy metal exposure is a significant public health problem, especially among children, who are a particularly vulnerable group. This study investigates the non-dietary exposure of children to lead, cadmium, and zinc and the associated health risk in three selected locations near the former non-ferrous metal smelters. Soil samples were collected from schools, parks, playgrounds, and other recreational places where children spend their free time in three districts of such towns as Katowice, Świętochłowice, and Piekary Śląskie. The contents of Cd, Pb, and Zn in the surface soil samples had the following ranges: 4.09–20.94 mg Cd/kg d.m., 161.70–1027.68 mg Pb/kg d.m., and 577.76–1475.93 mg Zn/kg d.m., respectively. The threshold doses of Cd, Zn, and Pb are 0.001 mg × kg^−1^ × day^−1^, 0.3 mg × kg^−1^ × day^−1^, and 0.0035 mg × kg^−1^ × day^−1^. A significant health risk was estimated as a result of non-dietary exposure of children to lead. The greatest non-cancer health risk in the population of children <6 years of age and in younger school children (<12 years of age) was shown. The problem was especially concerning in the scenario that assumed ingestion of soil particles in the areas most heavily contaminated with lead in the Katowice—Szopienice district. The public health policy should aim to monitor the current exposure of the local population to Pb and educate them on effective prophylactic methods to minimize environmental health risks.

## 1. Introduction

Environmental exposure to heavy metals such as cadmium (Cd), lead (Pb), and zinc (Zn) poses a significant health risk to the population. Exposure to these elements occurs primarily through the digestive tract. The inhalation route of exposure is much less important [1,2,3,4,5]. Children are mainly at particular health risk from exposure to heavy metals. The greater sensitivity of children versus adults results from such physiological conditions as lower bod-y weight, increased lung ventilation, higher metabolic rate, shorter height, and the related location of the nostrils at a lower height above the ground where the concentration of many pollutants is higher, consumption of a larger mass of food per kilogram of body weight, development of the immune system, formation of the blood-brain barrier, lower pH in the stomach, etc. In the case of small children, the most important source of exposure to lead and other heavy metals is the non-dietary route, including, among others, behaviors typical of this population group resulting from the lack of established hygiene habits and the tendency to put contaminated hands and objects into the mouth [6,7,8,9]. Non-dietary exposure of children to lead or cadmium is common in industrialized and urbanized areas. The problem is particularly noticeable in the areas affected by mining and processing plants of non-ferrous metal ores, landfills for waste originating from such plants, and areas where electronic waste is stored [10,11,12]. Exposure results from children swallowing dust or soil particles containing heavy metals. The threat is significant when soils are heavily contaminated with metals, as high doses of these pollutants can enter the body. The extent of children’s exposure to metals also depends largely on their diet, as the amount of calcium and other elements influencing the absorption of metals in the digestive tract is important [13]. Also important in this respect is the fact that children consume meals or snacks while playing on playgrounds, playing fields, or in yards. During consumption, soil particles found on the surface of dirty hands may be swallowed along with food [14]. Therefore, we should pay attention to the need to feed our children before going outside and to clean their hands regularly while playing. Health hazards posed by heavy metals commonly found in industrial and urban areas are associated with a wide range of negative health effects. For example, exposure to cadmium may cause, among others, kidney damage, atherosclerotic changes, skeletal dysfunctions, and malignant tumors, e.g., lung, prostate, kidney, and pancreatic cancer [15,16,17]. Exposure to cadmium during childhood may lead to growth retardation, as well as neurobehavioral and immunological disorders [18,19,20]. In turn, chronic exposure to lead, even at low concentrations, leads to irreversible damage to the central nervous system in children, manifested by a decrease in IQ, difficulties in learning, behavioral disorders, and problems with concentration, and it may also lead to speech and hearing disorders [17,18,21]. Lead also has a hepatotoxic effect, causes anemia, rickets, and lead colic, weakens male fertility, and disturbs calcium metabolism [17,22,23]. In turn, zinc supplied to the body in high doses disturbs the homeostasis of metal ions, may lead to immunosuppression and erythropoiesis disorders, and may also affect the metabolism of other microelements such as copper and iron, causing their deficiencies in the body [24,25,26]. 

The region of Poland with the highest environmental contamination with heavy metals is Upper Silesia. This area is historically associated with intensive industrial activity, including metallurgy and the extraction and processing of non-ferrous metal ores. As a result of many years of activity in these economic sectors, the natural environment in Upper Silesia has been polluted with cadmium, lead, zinc, and other metals [27,28]. Even though over the last few decades, due to intensive economic and systemic changes, the emission of these elements into the environment in the region has been significantly reduced, their concentrations, especially in soils, are still very high because soil is the place where metal compounds accumulate [29,30]. Historically, the largest emitters of heavy metals in Upper Silesia included plants such as the Bolesław Mining and Metallurgy Plant in Bukowno, the Non-Ferrous Metals Smelter in Katowice—Szopienice, and the Orzeł Biały Mining and Metallurgy Plant in Bytom.

The currently operating plant emitting significant amounts of metal-bearing dust into the environment is the zinc smelter “Miasteczko Śląskie” [31]. The administrative area of Upper Silesia covers a relatively small area of 12.3 thousand km^2^, constituting 3.9% of the area of Poland, and is inhabited by 4.4 million people, which translates into a high population density (357 people/km^2^ in 2021). Contamination of the local environment with heavy metals is therefore a source of exposure for a large population [32,33,34]. The groups at greatest health risk in society are the most vulnerable, including children, as well as residents with the lowest socio-economic status. Particular attention is paid to the problem of the highest exposure to heavy metals among children from the poorest districts of the cities of the industrial agglomeration of Upper Silesia, such as Lipiny in Świętochłowice, Szopienice in Katowice, and Brzeziny in Piekary Śląskie. The problem of health-threatening exposure of children to lead in the region was first noticed in the 1970s by Dr. Jolanta Wadowska-Król in the area affected by the Non-Ferrous Metal Smelter “Szopienice”. Thanks to her activity and persistence, the treatment of many children in sanatoriums was started, the purchase of milk was financed, the houses standing closest to the steelworks were demolished, etc. [35,36]. The above-mentioned districts of the cities of Upper Silesia are the focus of socio-economic problems resulting from the economic transformation that took place in the 1990s and was associated with the liquidation of large industrial plants employing the local population and a sharp increase in unemployment. The effects of those changes are still visible today, as the districts in question struggle with many problems, such as alcohol addiction, high crime rates, and joblessness. In the case of some children growing up in such conditions, unfortunately, the principles of hygiene and prevention of exposure to heavy metals were not taught from an early age. Therefore, assessing the exposure and health risks of children living in these areas is crucial for developing effective health prevention programs [37,38].

The research undertaken in this paper aimed to assess heavy metal contamination of playgrounds, primary school grounds, and their premises in Katowice—Szopienice, Świętochłowice—Lipiny, and Piekary Śląskie—Brzeziny, in the aspect of non-nutritional exposure of children to lead, cadmium, and zinc and the associated health risk. An additional aim of the work was to analyze the results of the eighth-grade examination of children in the studied schools compared with the results obtained in the entire Silesia Province and schools located in other districts of the towns under consideration.

## 2. Materials and Methods

### 2.1. Location of the Study Area

**Figure 1:**
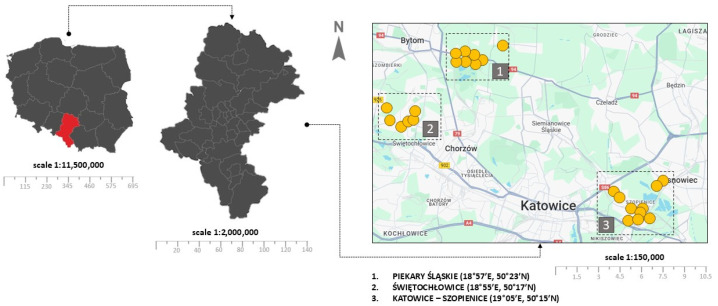
Location of the study area.

The research material consisted of soil samples collected from schools, parks, playgrounds, etc., in Katowice (KT)—Szopienice district, Świętochłowice (SW)—Lipiny district, and Piekary Śląskie (PS)—Brzeziny Śląskie district. A total of N = 77 soil samples were collected from playgrounds (N = 51), sports fields and gyms (N = 15), and other recreational places where children spend their free time (N = 11). Detailed characteristics of the sampling sites along with geolocation data are presented in Table S1 (Supplementary Material). The location of the study area is presented in Figure 1.

### 2.2. Sample Preparation

Soil samples from playing fields, playgrounds, gyms, and other recreational areas were collected in places where intensive dust generation from the ground occurs, i.e., near sports goals, swings, slides, and sandboxes. In the case of places where the surface was, for example, covered with grass or a polyurethane surface, samples were taken from the roadside. To prepare a representative 0.5 kg soil sample, several individual samples were taken from the topsoil (to a depth of 0.25 m) using a shovel and mixed thoroughly. The collected soil samples were placed in zip-lock bags. The samples prepared in this way were transferred to a cuvette, cleaned of visible stones and plant debris, and placed in a laboratory dryer SLW 75 SMART (POL-EKO-APARATURA, Wodzisław Śląski, Poland). The samples were dried at 105 °C until dry mass was obtained. To prepare a homogeneous sample, the dried soil samples were sieved through a sieve with a mesh size of <2 mm using an EML200 Pure HAVER&BOECKER (VWR, Oelde, Germany) sieve screen. From the prepared samples, a 0.5 g (±0.015 g) weight was prepared using a precision laboratory balance, model PS 750/X (RADWAG, Mazowieckie (Masovian) Voivodeship, Poland). The samples were transferred to a Teflon vessel to which 10 mL of 65% ultrapure nitric acid (Merck, North Rhine-Westphalia, Germany) and 1 mL of 30% hydrogen peroxide (Stanlab, Lublin, Poland) were added and then placed in a multi-station microwave mineralizer ETHOS UP (Milestone, Sorisole, Italy) and subjected to a three-stage mineralization process:

- Mineralization time: 20 min, temperature: 210 °C, generator power: 1800 W;
- Mineralization time: 15 min, temperature: 210 °C, generator power: 1800 W;
- Sample cooling: 30 min.

The mineralized soil samples were filtered into volumetric flasks and made up with ultrapure water to a previously determined volume.

### 2.3. Chemical Analysis of Heavy Metal Concentration in the Tested Samples

The concentrations of cadmium, lead, and zinc in the mineralized soil samples were determined by inductively coupled plasma optical emission spectroscopy (ICP-OES) using an ICP-OES Ultima Expert spectrometer from Horiba Scientific (Palaiseau, France). The studies were conducted in the Analytical Laboratory (AB1717) of the Department of Environmental Health, Faculty of Public Health in Bytom, Medical University of Silesia in Katowice, accredited by the Polish Centre for Accreditation (PCA). The obtained results were presented in mg/kg of dry mass (d.m.), and the limit of quantification (LOQ) was 0.18 mg/kg of dry mass for cadmium, 15.0 mg/kg of dry mass for lead, and 3.9 mg/kg of dry mass for zinc.

### 2.4. Quality Control and Quality Assurance

To maintain measurement traceability, certified reference materials (CRMs) were used to determine the calibration curve and to confirm the correctness of the measurements performed. To prepare the calibration curve for cadmium, lead, and zinc, certified reference materials from CPAchem (Bogomilovo, Bulgaria) were used at a concentration of 1000 mg/L in 2% HNO_3_ and with the following reference numbers: lead—C041.2NP.L1, cadmium—C010.2NP.L1, and zinc—C069.2NP.L1. The correctness of the conducted research was checked using the clay soil CRM (Trace Metals—Clay 1, CRM046-50G) manufactured by Sigma Aldrich (Laramie, WY, USA). The validation of the method is outlined in Table 1.

### 2.5. Assessment of Children’s Exposure and Health Risks

The non-dietary exposure of children to zinc, cadmium, and lead contained in soil and dust samples was estimated by calculating the average daily dose (ADD) of the metals using the formula recommended by the United States Environmental Protection Agency (US EPA) [39].

ADD = (MC × IR × CF)/BW,(1)

where the following are used:

*MC—Metal Concentration* (mg/kg dry mass).

*IR—Ingestion Rate by children* (mg/day).

*CF—Conversion Factor* (10^−6^ kg/mg).

*BW—Body weight* (kg).

The calculated doses of exposure of children were used to estimate the non-cancer health risk. For this purpose, the hazard quotient (HQ) was calculated using the following formula:

HQ = ADD/RfD,(2)

Reference dose (RfD) values for individual heavy metals were found in toxicology databases. The following RfD values were used in the calculations: 0.001 mg × kg^−1^ × day^−1^ for cadmium [40], 0.3 mg × kg^−1^ × day^−1^ for zinc [41]. Due to the lack of current recommendations for the reference dose for lead, a value of 0.0035 mg × kg^−1^ × day^−1^ [42] was calculated for this study by dividing the historically used PTWI (Provisional Tolerable Weekly Intake) by seven, which is a common practice in estimating the health risk of people exposed to lead (Table 2).

The risk assessment results were interpreted in such a way that if the value of the estimated hazard quotient was equal to or higher than 1 (HQ ≥ 1), the health risk to children was considered significant, and if HQ was less than 1 (HQ < 1), the result was interpreted as not posing a significant health risk. Additionally, the hazard index (HI) value was calculated by summing up the health risk posed by individual metals in total.

HI = HQ_Pb_ + HQ_Cd_ + HQ_Zn_, (3)

Values for HI lower than one (HI < 1.0) indicate that no potential negative impact on human health has to be considered and there are no impacts or risks of non-carcinogenic effects.

Because, in Poland, primary school is attended by children aged 7 to 15 years, the assessment of exposure and non-cancer health risk was carried out in two scenarios, taking into account children aged 6 to <12 years (younger children) and adolescents aged 12 to 15 years (older children). In each group, three variants of potential exposure were assumed:

- Scenario 1 (S1) assumes exposure of children to the lowest demonstrated concentrations of metals;
- Scenario 2 (S2) assumes exposure of children to average concentrations of the tested metals;
- Scenario 3 (S3) assumes exposure of children to the highest demonstrated metal concentrations.

To assess the exposure of children from the studied localities to heavy metals, the exposure indicator values recommended by the US EPA were used in the calculations [9]. The recommended body weight values do not coincide with the daily soil and dust ingestion values in the individual age groups [43]. Therefore, for this study, a lower body mass value was assumed for children aged 11–12 years, which, however, does not pose a risk of underestimating the research results. Similarly, in the case of preschool children, the recommended body weight for children aged 3 to <6 years was assumed, assuming the amount of soil consumption established for the age group from 1 to <6 years (Table 3).

An additional component of the present study comprised an evaluation of children’s inhalation and dermal exposure to heavy metals. The following formula was used to calculate the dermal exposure (the exposure factors used in the calculation [9,44,45] are shown in Table 4):

ADD = (C_soil_ × CF × SA × AF × ABS × EF × ED)/(BW × AT) (4)

where the following are used:

C—*Concentration of metal in soil* (mg/kg).

CF—*Conversion factor* (10^−6^ kg/mg).

AF—*Soil-to-skin adherence factor* (mg/cm^2^).

ABS—*Absorption factor* (unitless).

SA—*Skin surface area available for contact* (cm^2^/event).

EF—*Exposure frequency* (events/year).

ED—*Exposure duration* (years).

BW—*Body weight* (kg).

AT—*Averaging time* (days/years).

In order to calculate the inhalation exposure of children to metals, data from the National Environmental Monitoring (Chief Inspectorate of Environmental Protection) network were used [46]. The only station in the region that tests the concentration of lead and cadmium in particulate matter (PM_10_) is located in Katowice. However, it should be noted that the concentrations of zinc in these samples have not been tested. The mean annual values of Cd and Pb concentrations for the years 2023 and 2024 were obtained from the National Environmental Monitoring database. The quantity of inhalational exposure experienced by children was determined on these grounds. As demonstrated in Table 5, the calculations were based on the exposure factors [9,47] and heavy metal air contents. The following formula was used for calculations:

ADD = C_air_ × InhR/BW (5)

where the following are used:

C_air_—*Concentration of metal in PM_10_* (Cd ng/m^3^; Pb µg/m^3^).

InhR—*Inhalation Rate* (m^3^ day^−1^).

BW—*Body weight* (kg).

According to the US Environmental Protection Agency’s (EPA) Integrated Risk Information System (IRIS), a quantitative estimate of carcinogenic risk to lead (Pb), cadmium (Cd), and zinc (Zn) is not currently available. The values of CSF are not subject to assessment within the framework of the IRIS Program. Consequently, the carcinogenic risk was not calculated.

### 2.6. Obtaining Data on the School Achievements of Children in Contaminated Areas

Due to the neurotoxic effects of children’s exposure to lead proven in scientific literature, which results in, among others, a deterioration in school achievements, for this study, a comparison of the results of the eighth-grade exam was made among children from three studied schools, i.e., Primary School No. 42 in KT, Primary School No. 19 in SW, and Primary School No. 15 in PS. The results obtained in these schools in 2019, 2021, and 2024 were compared with provincial and national data. The eighth-grade exam is a compulsory exam taken in written form at the end of eight years of primary school. Children taking the exam are fifteen years old. The exam is a summary of the knowledge acquired throughout the education process. The basic terms take place in May and last three consecutive days. The exam paper contains closed and open-ended tasks. It comprises three subjects: Polish, mathematics, and a modern foreign language, the most frequently chosen of which is English. The Polish language exam lasts 120 min, the mathematics exam 100 min, and the foreign-language exam 90 min. The results for each subject separately are a percentage result for the percentage of points rounded to a whole number. Additionally, the result is also presented on a percentile scale. The results of the eighth-grade exam were obtained from publicly available databases of the Central Examination Commission (CEC) in Warsaw and the District Examination Commission (DEC) in Jaworzno [48,49]. The results were expressed as a percentage of the arithmetic mean, standard deviation, and median.

### 2.7. Statistical Analysis of Results

Statistical analysis was performed using Statistica version 13.3 software (StatSoft, Kraków, Poland). The normality of the distribution of variables was assessed using the Shapiro–Wilk test. Due to the lack of normality for the variables of cadmium, lead, and zinc concentration in soil samples (Shapiro–Wilk test: *p* < 0.05), nonparametric tests, i.e., Kruskal–Wallis ANOVA ranks and the Mann–Whitney U test, were used in further statistical analysis.

## 3. Results

### 3.1. Cd, Pb, and Zn Content in Soil Samples

The obtained results indicate varied heavy metal contamination of soils originating from playgrounds and recreational and sports facilities located in the studied cities. Polish legislation does not specify the maximum permissible concentrations of heavy metals in soils in children’s playgrounds. Therefore, reference was made to the maximum permissible concentration (MPC) specified in the Regulation of the Minister of Environment of 1 September 2016 on the method of assessing soil contamination, which in the case of cadmium, lead, and zinc is 2 mg Cd/kg d.m., 200 mg Pb/kg d.m., and 500 mg Zn/kg d.m., respectively [50]. Of the 77 soil samples collected, 65 of them (84.42%) exceeded the normative value for at least one of the three heavy metals analyzed. The MPC value was exceeded for Cd in 58 samples (75.32%), for Pb in 46 samples (59.74%), and for Zn in 44 samples (57.14%). In the case of 35 samples (45.45%), the determined concentrations exceeded the MPC for all three elements (Table S1—Supplementary Material).

The average concentrations of all three analyzed elements indicate that the most contaminated soil samples were collected from squares, parks, and green areas. The average concentrations of Cd, Pb, and Zn were 12.26 mg Cd/kg d.m., 554.51 mg Pb/kg d.m., and 1800.21 mg Zn/kg d.m., respectively. All average values exceeded the MPC, even several times, and the maximum concentrations even a dozen or so times. The lowest average concentrations of the analyzed metals, in the case of Cd and Zn, were found in playgrounds (7.10 mg Cd/kg d.m. and 799.92 mg Zn/kg d.m., respectively), while in the case of lead, in school playing fields or gyms (285.45 mg Pb/kg d.m.). Although these were the lowest average concentrations, these values also exceeded the MPC (Table 6). 

The obtained results, taking into account the three studied localities, indicate that the average contents of Cd, Pb, and Zn in samples taken from recreational areas are in the ranges of 4.09–20.94 mg Cd/kg d.m., 161.70–1027.68 mg Pb/kg d.m., and 577.76–1475.93 mg Zn/kg d.m., respectively. The most contaminated playgrounds with Cd and Pb are located in KT (7.75 mg Cd/kg d.m. and 554.51 mg Cd/kg d.m.), while in the case of Zn, the dominant average concentrations were recorded in PS (1077.80 mg Zn/kg d.m.). Soil samples taken from playing fields or gyms containing the most Cd and Pb came from PS (10.19 mg Cd/kg d.m. and 323.95 mg Pb/kg d.m.), while the most Zn was determined in samples from SW (1598.53 mg Zn/kg d.m.). The highest concentrations of all three analyzed elements were determined in soil samples collected from squares, parks, and green areas (category “other”) located in PS (20.94 mg Cd/kg d.m., 1027.68 mg Pb/kg d.m., and 2859.65 mg Zn/kg d.m., respectively) (Table 7). Table 6 and Table 7 also compare soil contamination with the FAO/WHO maximum permissible limits [51].

Despite the differences in the concentration of the tested metals in individual locations, the statistical analysis of the results did not confirm their significance (Kruskal–Wallis ANOVA ranks: *p* > 0.05) (Table 8). The probable cause is the large variation in contamination of the tested samples within individual categories, which also resulted in high values of standard deviations.

### 3.2. Children’s Non-Dietary Exposure and Health Risks

The conducted health risk assessment showed the greatest health risk in the youngest age group, i.e., in the population of children <6 years of age. A significant non-cancer health risk was estimated as a result of non-dietary exposure of children to lead. The problem concerned scenario S3, which assumed ingestion of soil particles in the areas most heavily contaminated with lead in Katowice—Szopienice. Lead exposure was more than three times the threshold dose. A lower, although still worrying, risk was shown in scenario S3 in Piekary Śląskie-Brzeziny, as the calculated HQ = 0.98, i.e., it was close to the threshold dose, the exceedance of which may be associated with the development of health disorders. In general, the average value of exposure of preschool children living in the studied localities of the Silesia Province to lead through the non-dietary route (S2) was about half of the reference dose, in the HQ range from 0.2 to 0.56, respectively, in SW–KT. Exposure of preschool children to cadmium and zinc was low enough not to pose a significant health risk (Table 9). The exposure of school children <12 years of age to the tested metals was significantly lower compared with preschool children. A significant health risk was found in the scenario assuming exposure to lead at the most contaminated playground in Katowice—Szopienice (HQ = 1.33) (Table 10). It is also worth noting the high, although lower than the reference dose, exposure to lead in Piekary Śląskie (HQ = 0.43). However, no significant non-cancer health risk was found as a result of exposure of younger school children to cadmium and zinc. In the group of the oldest school children, no health-threatening exposure to the tested metals was recorded, regardless of the exposure scenario considered and the place of residence. The highest hazard quotient value (HQ = 0.25) was shown in scenario S3, assuming non-dietary exposure of children to lead in Katowice—Szopienice. In turn, the non-dietary exposure of children aged 12–15 to cadmium and zinc was so low that it did not translate into a serious health risk (Table 11).

As part of this study, the total health risk resulting from the combined exposure of children to cadmium, lead, and zinc was also calculated. It was shown that the exposure of children from Upper Silesia to metals decreases with age. Therefore, the hazard index values can be ranked in the following order: preschool children > younger school children > older school children. Among preschool children in scenario S3, the hazard index value indicating significant health risk was shown in Katowice (HI = 3.15) and Piekary Śląskie (HI = 1.07). The lowest risk was estimated for preschool children in Świętochłowice (HI = 0.61). Taking into account the type of sites examined in general, the risk level can be ranked in the following order: playgrounds > other > playing fields. However, the order cited differed depending on the city studied (Table 12). In the group of school children aged 6 to <12 years, the combined exposure to cadmium, lead, and zinc results in a significant health risk in scenario S3 (HI = 1.38). A more detailed analysis showed that the problem concerned children spending their free time on playgrounds in Katowice. An increased index value (HI = 0.74) was estimated in scenario S3 in the areas classified as “other” in Katowice (Table 13). For older school children aged 12–15 years, no significant health risk was identified in any of the exposure scenarios analyzed. The highest index value (HI = 0.26) was estimated in scenario S3, and the highest exposure to metals occurred in the playground in Katowice—Szopienice (Table 14).

### 3.3. Children’s Dermal and Inhalation Exposure to Heavy Metals 

The estimated doses of cadmium, lead, and zinc that reached the bodies of children in Upper Silesia via the dermal route were found to be negligible, thereby confirming the insignificant role of this route in the total exposure to heavy metals (Table 15).

The findings of research conducted to date indicate that the inhalation exposure of children to cadmium and lead is at a low level. Nevertheless, the findings of this study demonstrate that the respiratory route accounts for greater exposure of children from Upper Silesia to heavy metals than the dermal route (Table 16).

### 3.4. School Achievements of Children from the Studied Areas Contaminated with Heavy Metals

Due to the proven neurotoxic properties of lead, the influence of this metal on the weakening of cognitive abilities, and the weakening of school results of children and adolescents, it was decided to compare the results of the state examination of children finishing primary school. The results of children attending three selected schools located in the studied districts of KT, SW, and PS were compared with the average for these three cities, for the Silesia administrative area, and with the national data. It was found that in 2019, 2021, and 2024, the results of the Polish language, mathematics, and English language exams among children from the analyzed primary schools in Katowice—Szopienice and Świętochłowice—Lipiny were significantly lower than the average results in these two cities, in Silesia Province, and in Poland. The poorest results were achieved in Primary School No. 19 in SW, located in the postindustrial district of Lipiny. The results of children attending school at PS are interesting compared with other schools. In 2019 and 2021, graduates of the school in the Brzeziny district achieved much better results than their peers from the entire city, region, and Poland, while in 2024, the average exam results were much lower (Table 17). The presented data on school achievements correspond with the demonstrated local problem of children’s non-dietary exposure to lead. It is therefore very likely that soil contamination with heavy metals in children’s recreation areas in the cities studied is, in addition to socio-economic conditions, one of the most important reasons for poor educational results among children.

## 4. Discussion

The research conducted as part of this work confirmed the relevance of the problem of soil contamination in recreation areas for children and youth in three postindustrial districts of cities located in the Silesia agglomeration. The average concentrations of cadmium, lead, and zinc exceeded the maximum permissible concentrations in all cities studied, regardless of the nature of the analyzed recreational area. Even though many years have passed since the cessation of operations of metallurgical plants, which were the main source of non-ferrous metal emissions into the environment, very high concentrations of heavy metals have currently been demonstrated in the topsoil. This situation may pose a significant health risk to the local community, especially children. The dynamic processes of industrial and energy transformation in the region, as well as the development of postindustrial areas for recreational and housing purposes, mean that the inhabitants of Silesia, and especially the youngest generations, are not aware of the existing threat. The youngest residents live in cities where the landscape is currently devoid of steelworks, mines, and other plants, as well as postindustrial dumps. Therefore, there may be a false sense of security when staying in recreational areas, e.g., playing fields, playgrounds, parks, or squares. In turn, soil contamination in such places may constitute a significant health risk factor as a result of non-dietary exposure. The results of studies conducted in three selected cities show that the biggest problem currently is the exposure of children to lead. The doses of this metal entering the body through ingested soil particles are so high that they exceed safe exposure thresholds. This study confirmed that the younger the children, the greater the health risk. The most exposed and at the same time the highest health risk concerned preschool children, followed by younger school children >12 years of age. The strongest exposure may occur on playgrounds located in the Szopienice district of Katowice. In the youngest age group of children < 6 years, exposure to lead was more than three times higher than the reference dose. The findings of this research suggest that the inhalation and dermal exposure of children to metals pose negligible non-cancer risk. The relative proportions of particular exposure pathways were as follows: ingestion > inhalation > dermal. The demonstrated health risk associated with non-dietary exposure of children is the result of high concentrations of the tested metals in the most contaminated sites. A significantly lower, negligible risk was identified in the study by Mohammadi et al. (2020) conducted in the Khayyam industrial zone in Iran [52], where cadmium and lead soil concentrations were lower than in Upper Silesia. Similarly, children in kindergartens and parks in Bratislava (Slovakia) were also much less exposed to cadmium, lead, zinc, and other metals. As a result, the hazard index was below the threshold values in all the locations studied [53]. The highest non-cancer risk of heavy metals among different age groups in the coastal industrial region of the Yangtze River Delta, focusing on children. The inhabitants experienced the greatest exposure to As and Pb. The maximum HQ values for children in case of As, Pb, Cd, and Zn exposure were as follows: 2.03, 0.57, 0.078, and 0.02, respectively [54]. This result indicates a local problem of soil contamination with arsenic, and, to a lesser extent, with metals that are most commonly occurring in Upper Silesia. Penteado et al. (2021) conducted research in urban parks in Rio Grande City, southern Brazil, which revealed elevated non-carcinogenic risk concerns for children who use recreational spaces due to exposure to lead and, to a lesser extent, mercury [55]. These results indicate that exposure of children to heavy metals in urban and industrialized areas is a global public health problem. The magnitude of the problem is defined by the degree of environmental pollution and the type of metals present. As demonstrated in the case study of Upper Silesia, the health risks to children in this region are significantly elevated in comparison to other areas that have been documented in the existing literature as having challenges with exposure to heavy metals.

### 4.1. Soil Contamination with Heavy Metals in the Central Part of Upper Silesia Province as a Significant Environmental Health Risk Factor for the Local Population

The problem of soil contamination with heavy metals in the cities of the Silesia agglomeration is confirmed by the results of studies by other authors. For example, in the work of Piekut et al. [27], the normative concentrations of at least one of the tested metals (cadmium, lead, zinc) were exceeded in one-third of the analyzed samples from playgrounds and sports fields in Silesia cities such as Sosnowiec and Bytom. The estimated non-dietary exposure of children to the analyzed contaminants posed a significant health risk [27]. Moreover, the highest permissible concentrations of cadmium, lead, and zinc were also found to be exceeded in soils from residential estates, kindergartens, school playing fields, sandboxes, playgrounds, and lawns in various cities of the Silesia agglomeration [56,57]. Contamination of arable land in Silesia, where edible plants are grown, with heavy metals was found in the studies by Ćwieląg-Drabek et al. [58] and Dziubanek et al. [59]. Therefore, the population of Silesia residents, including children, may experience exposure to heavy metals also through the digestive tract. Therefore, the total doses of metals reaching the body as a result of combined exposure occurring through individual routes may be hazardous to the health of the local community. Currently, the only active non-ferrous metal ore processing plant in the Silesia Province is the zinc smelter in Miasteczko Śląskie. The plants are located north of the Silesia agglomeration, which means that densely populated cities are not within the direct range of the smelter’s impact. The results of Kicińska et al.’s research [60] indicate that the current contamination of soils with heavy metals in this region is much higher than the values recorded in the same places 20 years ago. These results confirm that the process of metal accumulation in the environment is still ongoing [60]. In turn, the studies conducted in these areas by Niezgoda et al. [61] showed that the consumption of mushrooms and blueberries from the surrounding forests may be associated with a significant health risk for both children and adults. Exposure to lead in the Upper Silesia region is confirmed by the results of measurements of the content of this metal in blood conducted in the years 1999–2013 in a population of 4882 children aged 3 to 18, living in twelve cities of the Silesia agglomeration. Studies have shown that the average blood lead level (BLL) in children was 28.67 µg/L, with the concentration range reaching 6.0–360.0 µg/L. It has also been shown that the factors influencing the increased level of lead in the blood of children included: lower level of education of parents, unemployment, occupational exposure of parents to lead, poor socio-economic status of the family, smoking at home, living on the ground floor of buildings, eating locally grown vegetables and fruits, spending longer time playing outdoors in a polluted environment, and male gender [62]. Increased exposure of children to heavy metals is a significant public health problem in many countries around the world. This applies to both developed and developing countries. According to the results of a study by Zhao et al. [63] conducted near mining areas in Chenzhou and Hengyang cities, located in the Hunan Province of China, the main source of population exposure to lead was accidental ingestion of household dust, while the most important source of exposure of the local community to arsenic and cadmium was the consumption of contaminated rice [63]. A significant non-cancer health risk has also been estimated in the population of children exposed to lead from soils from playgrounds and public parks in the capital of Ghana, Accra. Soil contamination in Ghana is primarily due to the long-term use of leaded fuel before its complete ban on 1 January 2004 [64]. In the U.S., an attempt has been made to develop an action plan to reduce children’s exposure to lead [65]. The concentrations of lead in soil in residential areas and dust that could result in BLL for children aged 1 to <2 years and 2 to <6 years were estimated at 5 μg/dL and 3.5 μg/dL, respectively. It was shown that depending on the age group, population percentile, and assumed BLL, the permissible lead concentrations in soils ranged from 70 mg/kg to 220 mg/kg. In the case of dust, the permissible lead concentrations ranged from 110 mg/kg to 240 mg/kg [65]. These types of preventive measures are important because they protect children from negative health effects, especially neurotoxic effects, which are measured by reduced intelligence quotient (IQ). The average global IQ decline attributable to dietary lead exposure in 2015 was estimated to be 1.1. The total disability-adjusted life years (DALYs) attributable to IQ reduction were estimated at 5.2 million DALYs, with an uncertainty range of 0–31 million DALYs [66]. 

### 4.2. Summary of Findings and Recommendations

The results of state examinations presented in this paper among children finishing education in selected primary schools in postindustrial districts of cities in the Silesia agglomeration are, in most cases, much lower than the average number of points obtained in individual cities, in the province, and the country. On this basis, it can be assumed that one of the determinants of this situation may be the problem of the increased exposure of children from the studied districts to lead. In this context, it is necessary to implement preventive measures. First of all, educational activities should be implemented, aimed at both parents and children themselves. Residents of areas burdened with postindustrial pollution should develop habits in their daily lives that will minimize exposure to lead, e.g., hand hygiene, frequent washing of toys, furniture, floors, and objects with which children come into contact, and avoiding eating locally grown edible plants. Local government representatives should ensure children’s safety on sports fields, playgrounds, and other recreational areas, e.g., by securing surfaces against secondary dusting, frequent replacement of sand in sandboxes, etc. [67,68]. Moreover, it is also advisable to monitor the concentration of heavy metals in soils in places where children spend time [69]. The effects of protective measures can bring many social and financial benefits, as evidenced by the experience of the Flanders region in Belgium in the prevention of lead exposure [70]. The implemented actions allowed saving as much as EUR 7176 million in social costs related to the reduction in IQ loss over 15 years [70]. In connection with the above, and in light of the results presented in this work, it is extremely important to regularly remind the society of Upper Silesia about the problem of population exposure to heavy metals and the related health risk, especially for children. It is unfortunate that the local and governmental authorities in Poland currently lack awareness of the gravity of the issue. The monitoring of BLL in children across the Upper Silesia region is exclusively conducted by the ‘Miasteczko Śląskie’ Foundation for Children, a non-governmental organization. The Foundation also develops educational materials, which are primarily distributed in local schools. It is also necessary to monitor educational, recreational, and residential facilities, as well as to include content on the prevention of exposure to heavy metals in school and kindergarten curricula. In addition, social campaigns dedicated to the general population should be implemented to remind people about the problem and to indicate simple measures for managing health risks at an individual level. Due to the nature of the problem, research on the exposure of children from postindustrial districts of the Silesia agglomeration to lead and other heavy metals is being continued. The next work being prepared will present the results of the exposure of school children to metals contained in dust found inside and outside the premises of local primary schools. 

## 5. Conclusions

This study assessed the health risk of lead, cadmium, and zinc exposure of preschool and school children from postindustrial districts of the Silesia Province. The elevated concentrations of metals, which exceed the maximum permissible concentrations, on the surfaces of sports fields, playgrounds, and other recreational areas have been identified as a contributing factor to the non-dietary exposure to lead in preschool children (aged <6 years) and younger school children (aged <12 years). This exposure has been associated with a significant non-cancer health risk. In the scenario where children are exposed to the highest demonstrated metal concentrations, the risk among preschool children is very high, with a measured hazard quotient (HQ) of 3.12. Children’s dermal and inhalation exposure to heavy metals was much lower, indicating the particular role of non-nutritional exposure in postindustrial areas like Upper Silesia.

Significantly lower results in state examinations at the end of primary school education were observed among students in schools located in postindustrial districts of cities in the Silesia agglomeration, which may be due, among other things, to increased exposure of children to lead. It is necessary to continue monitoring the quality of the living environment of the society in Upper Silesia in terms of heavy metal pollution. Education to prevent exposure to heavy metals from local sources should also be provided to both children and adults.

## Figures and Tables

**Table 1:** Validation of the research method.

Characteristics of the Method	ICP-OES		
Elements	Zn	Pb	Cd
Line [nm]	206.201	220.353	228.802
Linearity—correlation coefficient: R^2^ ≥ 0.995	0.999	0.999	0.999–1.000
Accuracy—RSD < 10% standards	0.62–2.50	0.49–4.30	0.37–2.90
Repeatability—R < 20%	1.5–6.49	0.84–8.21	1.75–18.57
LOQ [mg/kg]	3.28	12.68	0.15
LOD [mg/kg]	1.43	5.80	0.06
Recovery CRM (Trace Metals—Silt Clay 1; CRM045-50G) [75–125%]	81–105	92–118	87–99

**Table 2:** The reference dose values used in the present study.

Heavy Metals	Reference Dose (RfD)
Cadmium	0.001 mg × kg^−1^ × day^−1^
Lead	0.0035 mg × kg^−1^ × day^−1^
Zinc	0.3 mg × kg^−1^ × day^−1^

**Table 3:** Exposure factors used in the study.

Exposure Factors	1 to <6 Years	6 to <12 Years	12–15 Years
Soil ingestion (mg/day)	40	30	10
Body weight (kg)	18.6 kg *	31.8 kg **	56.8 kg ***

* recommended for 3 to <6 years. ** recommended for 6 to <11 years. *** recommended for 11 to <16 years.

**Table 4:** The exposure factors used in the dermal exposure assessment.

Factors	Preschool Children	Younger School Children	Older School Children
Soil-to-skin adherence factor (mg/cm^2^)	0.04		
Absorption factor of metals	0.001		
Skin surface area available for contact (cm^2^/event)	Boys: 0.728 Girls: 0.711	Booth gender: 1.16	Boys: 1.49 Girls: 1.48
Exposure frequency (events/year)	365/year		
Exposure duration (years)	1 year		
Body weight (kg)	18.6	31.8	56.8
Averaging time (days/years)	365 days/year		

**Table 5:** The exposure factors used in the inhalation exposure assessment.

Factors	Preschool Children	Younger School Children	Older School Children
InhR (m^3^/d)	10.1	12.0	15.2
BW (kg)	18.6	31.8	56.8
Annual air concentration of heavy metals	2023	2024	
	Cd (ng/m^3^)		
Min	0.10	0.10	
$\bar{x}$	0.63	0.76	
MAX	1.71	4.72	
	Pb (µg/m^3^)		
Min	0.001	0.003	
$\bar{x}$	0.025	0.021	
MAX	0.094	0.083	

**Table 6:** Heavy metal content in soil samples from urban squares, playgrounds, gyms, sports fields, and primary school surroundings.

Recreational Area	N Samples	Concentration of Toxic Elements (mg/kg d.m.)														
		Cd					Pb					Zn				
		MIN	MAX	$\bar{x}$	SD	ME	MIN	MAX	$\bar{x}$	SD	ME	MIN	MAX	$\bar{x}$	SD	ME
playground	51	0.23	30.40	7.10	7.59	3.51	7.50	4930.10	379.33	737.16	160.84	43.66	3528.37	799.92	722.34	519.44
field/gym	15	1.06	40.25	8.91	9.99	7.48	44.82	952.77	285.45	242.64	235.18	123.87	4743.33	1166.61	1193.50	1107.36
other	11	2.14	27.97	12.26	9.59	10.27	85.10	1594.04	554.51	470.24	470.24	353.92	4616.60	1800.21	1516.22	1454.10
all samples	77	0.23	40.25	8.19	8.47	4.86	7.50	4930.10	386.07	635.28	196.85	43.66	4743.33	1014.25	1016.36	720.65
MPC		2.00					200.00					500.00				
FAO/WHO		3.00					100.00					300.00				

MPC—maximum permissible concentration. FAO/WHO—Maximum Permissible Limits of FAO/WHO. $\bar{x}$—arithmetic mean. MIN—minimum value. MAX—maximum value. ME—median. SD—standard deviation.

**Table 7:** Content of heavy metals in soil samples in individual cities depending on the nature of the recreational area.

Recreational Area	Location	N Samples	Concentration of Toxic Elements (mg/kg d.m.)														
			Cd					Pb					Zn				
			MIN	MAX	$\bar{x}$	SD	ME	MIN	MAX	$\bar{x}$	SD	ME	MIN	MAX	$\bar{x}$	SD	ME
playground	KT	24	0.43	30.40	8.50	8.76	3.61	19.86	4930.10	513.67	1019.67	181.26	59.19	2462.85	778.99	656.69	539.36
	SW	14	0.23	13.96	4.09	4.30	2.32	7.50	658.42	161.70	173.47	121.07	43.66	2028.63	577.76	555.69	480.79
	PS	13	0.72	22.73	7.75	7.62	5.38	23.98	1334.00	365.70	383.56	266.53	50.43	3528.37	1077.80	933.22	773.72
field/gym	KT	9	1.06	40.25	8.46	12.59	3.54	44.82	952.77	274.10	293.97	183.20	123.87	4743.33	1007.44	1475.93	373.01
	SW	3	6.36	11.92	8.95	2.80	8.58	196.85	407.19	280.97	111.31	238.88	1126.73	2022.95	1598.53	449.99	1645.90
	PS	3	2.90	17.45	10.19	7.27	10.21	110.29	561.17	323.95	226.36	300.40	338.93	1942.52	1212.23	811.30	1355.22
other	KT	2	5.25	18.18	11.72	9.15	11.72	221.75	762.23	491.99	382.18	491.99	750.49	2192.23	1471.36	1019.47	1471.36
	SW	6	2.14	24.51	8.11	8.53	4.21	85.10	850.20	338.76	353.59	139.44	353.92	4616.60	1380.19	353.59	139.44
	PS	3	11.57	27.97	20.94	8.44	23.28	735.11	1594.04	1027.68	490.56	753.91	1454.10	4159.05	2859.65	1355.59	2965.81
all samplesin the town	KT	35	0.43	40.25	8.67	9.60	3.81	19.86	4930.10	450.83	859.73	199.88	59.19	4743.33	877.30	931.08	594.40
	SW	23	0.23	24.51	5.77	5.73	3.56	7.50	850.20	223.45	232.13	139.25	43.66	4616.60	920.21	1007.75	509.96
	PS	19	0.72	27.97	10.22	8.72	5.60	23.98	1594.04	463.63	440.13	282.31	50.43	4159.05	1380.37	1137.30	1345.92
MPC			2.00					200.00					500.00				
FAO/WHO			3.00					100.00					300.00				

KT—Katowice—Szopienice, SW—Świętochłowice—Lipiny, PS—Piekary Śląskie—Orzeł Biały. MPC—maximum permissible concentration. FAO/WHO—Maximum Permissible Limits of FAO/WHO. $\bar{x}$—arithmetic mean. MIN—minimum value. MAX—maximum value. ME—median. SD—standard deviation.

**Table 8:** Results of statistical analysis of heavy metal concentration variation in the tested soil samples depending on the sampling location.

Dependencies Studied	*p*-Value			
	Cadmium	Lead	Zinc	Test Used
KT vs. SW vs. PS	0.26	0.13	0.12	KW
playgrounds vs. field/gym vs. other	0.10	0.14	0.05	KW
playgrounds (KT vs. SW vs. PS)	0.21	0.24	0.19	KW
field/gym (KT vs. SW vs. PS)	0.43	0.63	0.15	KW
other (KT vs. SW vs. PS)	0.13	0.16	0.33	KW

KT—Katowice. SW—Świętochłowice. PS—Piekary Śląskie. KW—Kruskal–Wallis test.

**Table 9:** The non-dietary exposure to heavy metals and health risks of preschool children.

Metals/Samples from All Types of Locations	Scenario	Total Samples		KT		SW		PS	
		Dose (µg/kg/Day)	HQ	Dose (µg/kg/Day)	HQ	Dose (µg/kg/Day)	HQ	Dose (µg/kg/Day)	HQ
Cd	S1	0.0005	0.0005	0.0009	0.0009	0.0005	0.0005	0.0015	0.0015
	S2	0.0176	0.0176	0.0186	0.0186	0.0124	0.0124	0.0220	0.0220
	S3	0.0866	0.0866	0.0866	0.0866	0.0527	0.0527	0.0602	0.0602
Pb	S1	0.0161	0.0046	0.0427	0.0122	0.0161	0.0046	0.0516	0.0147
	S2	0.8303	0.2372	0.9695	0.2770	0.4805	0.1373	0.9971	0.2849
	S3	10.6024	3.0292	10.6024	3.0292	1.8284	0.5224	3.4280	0.9794
Zn	S1	0.0960	0.0003	0.1273	0.0004	0.0939	0.0003	0.1085	0.0004
	S2	2.1812	0.0073	1.8867	0.0063	1.9789	0.0066	2.9685	0.0099
	S3	10.2007	0.0340	10.2007	0.0340	9.9282	0.0331	8.9442	0.0298

KT—Katowice. SW—Świętochłowice. PS—Piekary Śląskie.

**Table 10:** The non-dietary exposure to heavy metals and health risks of younger school children.

Metals/Samples from All Types of Locations	Scenario	Total Samples		KT		SW		PS	
		Dose (µg/kg/Day)	HQ	Dose (µg/kg/Day)	HQ	Dose (µg/kg/Day)	HQ	Dose (µg/kg/Day)	HQ
Cd	S1	0.0002	0.0002	0.0004	0.0004	0.0002	0.0002	0.0007	0.0007
	S2	0.0077	0.0077	0.0082	0.0082	0.0054	0.0054	0.0096	0.0096
	S3	0.0380	0.0380	0.0380	0.0380	0.0231	0.0231	0.0264	0.0264
Pb	S1	0.0071	0.0020	0.0187	0.0054	0.0071	0.0020	0.0226	0.0065
	S2	0.3642	0.1041	0.4253	0.1215	0.2108	0.0602	0.4374	0.1250
	S3	4.6510	1.3289	4.6510	1.3289	0.8021	0.2292	1.5038	0.4297
Zn	S1	0.0421	0.0001	0.0558	0.0002	0.0412	0.0001	0.0476	0.0002
	S2	0.9568	0.0032	0.8276	0.0028	0.8681	0.0029	1.3022	0.0043
	S3	4.4748	0.0149	4.4748	0.0149	4.3553	0.0145	3.9236	0.0131

KT—Katowice. SW—Świętochłowice. PS—Piekary Śląskie.

**Table 11:** The non-dietary exposure to heavy metals and health risks of older school children.

Metals/Samples from All Types of Locations	Scenario	Total Samples		KT		SW		PS	
		Dose (µg/kg/Day)	HQ	Dose (µg/kg/Day)	HQ	Dose (µg/kg/Day)	HQ	Dose (µg/kg/Day)	HQ
Cd	S1	0.00004	0.00004	0.00008	0.00008	0.00004	0.00004	0.00013	0.00013
	S2	0.00144	0.00144	0.00153	0.00153	0.00101	0.00101	0.00179	0.00179
	S3	0.00709	0.00709	0.00709	0.00709	0.00432	0.00432	0.00492	0.00492
Pb	S1	0.0013	0.0004	0.0035	0.0010	0.0013	0.0004	0.0042	0.0012
	S2	0.0680	0.0194	0.0794	0.0227	0.0393	0.0112	0.0816	0.0233
	S3	0.8680	0.2480	0.8680	0.2480	0.1497	0.0428	0.2806	0.0802
Zn	S1	0.00786	0.00003	0.01042	0.00003	0.00769	0.00003	0.00888	0.00003
	S2	0.17857	0.00060	0.15445	0.00051	0.16201	0.00054	0.24302	0.00081
	S3	0.83509	0.00278	0.83509	0.00278	0.81278	0.00271	0.73223	0.00244

KT—Katowice. SW—Świętochłowice. PS—Piekary Śląskie.

**Table 12:** Total non-cancer health risk of preschool children non-dietary exposed to Cd, Pb, and Zn.

Location	Scenario	Hazard Index (HI)			
		Total Samples	KT	SW	PS
samples from all types of locations	S1	0.113	0.014	0.005	0.017
	S2	0.262	0.302	0.156	0.317
	S3	3.150	3.150	0.608	1.069
playground	S1	0.005	0.014	0.005	0.017
	S2	0.254	0.339	0.112	0.256
	S3	3.120	3.112	0.449	0.894
field/gym	S1	0.031	0.031	0.143	0.076
	S2	0.203	0.194	0.203	0.230
	S3	0.706	0.706	0.290	0.396
other	S1	0.059	0.153	0.059	0.487
	S2	0.380	0.338	0.235	0.697
	S3	1.073	0.523	0.608	1.069

KT—Katowice. SW—Świętochłowice. PS—Piekary Śląskie.

**Table 13:** Total non-cancer health risk of younger school children non-dietary exposed to Cd, Pb, and Zn.

Location	Scenario	Hazard Index (HI)			
		Total Samples	KT	SW	PS
samples from all types of locations	S1	0.049	0.006	0.002	0.007
	S2	0.115	0.132	0.069	0.139
	S3	1.382	1.382	0.267	0.469
playground	S1	0.002	0.006	0.002	0.007
	S2	0.111	0.149	0.049	0.109
	S3	1.369	1.365	0.197	0.392
field/gym	S1	0.013	0.013	0.063	0.034
	S2	0.089	0.085	0.089	0.101
	S3	0.310	0.310	0.127	0.174
other	S1	0.026	0.217	0.026	0.214
	S2	0.167	0.480	0.103	0.306
	S3	0.471	0.743	0.267	0.469

KT—Katowice. SW—Świętochłowice. PS—Piekary Śląskie.

**Table 14:** Total non-cancer health risk of older school children non-dietary exposed to Cd, Pb, and Zn.

Location	Scenario	Hazard Index (HI)			
		Total Samples	KT	SW	PS
samples from all types of locations	S1	0.0092	0.0011	0.0004	0.0014
	S2	0.0215	0.0247	0.0128	0.0259
	S3	0.2579	0.2579	0.0498	0.0875
playground	S1	0.0004	0.0011	0.0004	0.0014
	S2	0.0208	0.0278	0.0092	0.0204
	S3	0.2554	0.2548	0.0368	0.0732
field/gym	S1	0.0025	0.0025	0.0117	0.0063
	S2	0.0166	0.0159	0.0166	0.0188
	S3	0.0578	0.0578	0.0238	0.0324
other	S1	0.0049	0.0125	0.0049	0.0399
	S2	0.0311	0.0277	0.0193	0.0571
	S3	0.0878	0.0428	0.0498	0.0875

KT—Katowice. SW—Świętochłowice. PS—Piekary Śląskie.

**Table 15:** Range of the daily dose of the dermal exposure to heavy metals of children living in the central part of the Upper Silesia region.

	Cd µg/kg/d	Pb µg/kg/d	Zn mg/kg/d
Preschool children	3.5 × 10^−10—^6.3 × 10^−8^	1.15 × 10^−8^–7.72 × 10^−6^	6.83 × 10^−11^–7.43 × 10^−9^
Younger school children	3.3 × 10^−10^–5.87 × 10^−8^	1.09 × 10^−8^–7.19 × 10^−6^	6.52 × 10^−11^–6.92 × 10^−9^
Older school children	2.4 × 10^−10^–4.2 × 10^−8^	7.82 × 10^−9^–5.17 × 10^−6^	4.65 × 10^−11^–4.98 × 10^−9^

**Table 16:** Inhalation exposure to heavy metals of children from Upper Silesia in the years 2023 and 2024.

	Cd ng/kg/d		Pb µg/kg/d	
Years	2023	2024	2023	2024
Preschool children				
Min	0.054	0.054	0.0005	0.002
$\bar{x}$	0.344	0.411	0.014	0.011
MAX	0.928	2.563	0.051	0.045
Younger school children				
Min	0.038	0.038	0.0004	0.001
$\bar{x}$	0.239	0.285	0.009	0.008
MAX	0.645	1.781	0.036	0.031
Older school children				
Min	0.027	0.027	0.0003	0.001
$\bar{x}$	0.170	0.202	0.007	0.005
MAX	0.458	1.263	0.025	0.022

**Table 17:** Results of the eighth-grade exam in 2024, 2021, and 2019.

City	Subject Exam		
	Polish Language $\bar{x}$ ± SD (ME) (%)	Mathematica $\bar{x}$ ± SD (ME) (%)	English Language $\bar{x}$ ± SD (ME) (%)
	**The Year 2024**		
Katowice (N = 55)	61.71 ± 22.39 (67)	57.31 ± 29.89 (60)	73.9 ± 28.12 (28.1)
Katowice—Szopienice PSC No. 42	51.52 ± 18.35 (53)	31.36 ± 25.32 (20)	45.04 ± 28.49 (33)
Świętochłowice (N = 7)	50.44 ± 21.86 (52)	40.23 ± 28.53 (32)	58.69 ± 31.41 (58)
Świętochłowice—Lipiny PSC No. 19	31.1 ± 14.5 (36)	22.1 ± 16.64 (16)	43.38 ± 29.5 (27)
Piekary Śląskie (N = 10)	52.14 ± 22.71 (56)	42.54 ± 27.84 (36)	63.31 ± 30.53 (69)
Piekary Śląskie—Brzeziny PSC No. 15	44.4 ± 25.23 (45.5)	20.8 ± 17.87 (16)	45.82 ± 31.74 (27)
Silesia Province	58.00 *	49.54 *	66.48 *
Poland	61 ± 21 (64)	52 ± 29 (48)	66 ± 30 (76)
	the year 2021		
Katowice (N = 51)	64.79 ± 18.84 (69)	51.17 ± 27.04 (48)	72.37 ± 28.34 (85)
Katowice—Szopienice PSC No. 42	42.94 ± 19.6 (43)	29.67 ± 23.71 (24)	42.94 ± 29.61 (28)
Świętochłowice (N = 8)	57.31 ± 20.95 (60)	40.38 ± 23.85 (36)	59.93 ± 30.11 (64)
Świętochłowice—Lipiny PSC No. 19	33.49 ± 17.86 (27)	21.77 ± 13.12 (20)	33.12 ± 24.33 (22)
Piekary Śląskie (N = 11)	56.57 ± 19.95 (60)	42.62 ± 24.27 (36)	62.28 ± 29.59 (67)
Piekary Śląskie—Brzeziny PSC No. 15	71.8 ± 15.06 (78)	49.2 ± 21.91 (50)	60.36 ± 30.83 (51)
Silesia Province	60.57 *	46.74 *	66.98 *
Poland	60 ± 19 (62)	47 ± 26 (44)	66 ± 29 (73)
	the year 2019		
Katowice (N = 59)	67.68 ± 20.09 (72)	48.69 ± 26.68 (47)	67.01 ± 28.68 (77)
Katowice—Szopienice PSC No. 42	47.06 *	23.80 *	41.45 *
Świętochłowice (N = 9)	63.81 ± 19.84 (68)	42.55 ± 24.74 (40)	56.11 ± 29.12 (55)
Świętochłowice—Lipiny PSC No. 19	42.13 *	22.06 *	25.67 *
Piekary Śląskie (N = 10)	63.37 ± 20.64 (68)	43.17 ± 26.07 (37)	57.73 ± 28.43 (58)
Piekary Śląskie—Brzeziny PSC No. 15	70.82 *	50.24 *	66.76 *
Silesia Province	64.13 *	44.74 *	60.24 *
Poland	63 ± 20 (66)	45 ± 26 (40)	59 ± 29 (60)

Source: Own elaboration based on [48,49]. $\bar{x}$ ± SD (ME)—arithmetic mean, standard deviation, median. PSC—primary school. * Databases do not include all sub-data (hence missing SD values).

## Data Availability

The data presented in this study are available upon request from the corresponding author. The data are not publicly available due to privacy restrictions.

## Supplementary Materials

The following supporting information can be downloaded at https://www.mdpi.com/article/10.3390/toxics13050377/s1, Table S1: Characteristics of soil sampling sites (numbered).

## Abbreviations

The following abbreviations are used in this manuscript:

ADD	Average daily dose
BW	Body weight
CEC	Central Examination Commission
CF	Conversion Factor
DALYs	Disability-adjusted life years
DEC	District Examination Commission
d.m.	Dry mass
HI	Hazard Index
HQ	Hazard quotient
ICP-OES	Inductively coupled plasma optical emission spectroscopy
IQ	Intelligence quotient
IR	Ingestion Rate by children
KT	Katowice—Szopienice
KW	Kruskal–Wallis test
LOQ	Limit of quantification
MAX	Maximum value
MC	Metal Concentration
ME	Median
MIN	Minimum value
MPC	Maximum permissible concentration
PCA	Polish Centre for Accreditation
PS	Piekary Śląskie—Orzeł Biały
PSC	Primary School
PTWI	Provisional Tolerable Weekly Intake
RfD	Reference dose
SD	Standard deviation
SW	Świętochłowice—Lipiny
US EPA	United States Environmental Protection Agency
$\bar{x}$	Arithmetic mean
