# Supplementary material for: Health Risk for Non-Dietary Children’s Exposure to Heavy Metals in Postindustrial Areas in Upper Silesia, Poland
Source: Toxics. 2025 May 6;13(5):377. doi: 10.3390/toxics13050377 (PMC12115573; doi:10.3390/toxics13050377)
Supplement: Supplementary file 1 [file toxics-13-00377-s001.zip › toxics-3553252-supplementary.pdf]

Supplementary Material

Table S1. Characteristics of soil sampling sites

Number of sample	Sample code	Location*	Recreational area**	Concentration of toxic element [mg/kg d.m.]		
				Cd	Pb	Zn
1	K69 II 22 23	KT	other	5.25	221.75	750.49
2	K84 II 22 23	KT	other	18.18	762.23	2192.23
3	K91 II 22 23	SW	other	3.30	86.26	366.67
4	K92 II 22 23	SW	other	3.56	114.29	424.45
5	K93 II 22 23	SW	other	2.14	85.10	353.92
6	K102 II 22 23	SW	other	4.86	164.59	1000.00
7	K111 II 22 23	SW	other	10.27	732.14	1519.02
8	K112 II 22 23	SW	other	24.51	850.20	4616.60
9	K115 II 22 23	PS	other	27.97	735.11	2965.81
10	K116 II 22 23	PS	other	23.28	1594.04	4159.05
11	K130 II 22 23	PS	other	11.57	753.91	1454.10
12	K58 II 22 23	KT	playground	13.03	1296.96	2462.85
13	K59 II 22 23	KT	playground	14.57	1551.84	1128.80
14	K60 II 22 23	KT	playground	3.81	396.36	1269.51
15	K61 II 22 23	KT	playground	26.32	4930.10	255.31
16	K62 II 22 23	KT	playground	0.43	19.86	1403.88
17	K63 II 22 23	KT	playground	2.08	102.93	68.45
18	K65 II 22 23	KT	playground	1.57	19.86	233.39
19	K66 II 22 23	KT	playground	2.28	93.10	279.51
20	K67 II 22 23	KT	playground	14.50	268.44	341.70
21	K68 II 22 23	KT	playground	10.00	199.88	813.63
22	K70 II 22 23	KT	playground	10.15	328.36	594.40
23	K71 II 22 23	KT	playground	3.28	122.13	1161.79
24	K72 II 22 23	KT	playground	0.79	21.84	484.32
25	K73 II 22 23	KT	playground	1.41	49.51	92.75
26	K74 II 22 23	KT	playground	19.25	771.75	200.10
27	K77 II 22 23	KT	playground	0.66	20.12	1965.98
28	K78 II 22 23	KT	playground	21.92	601.94	59.19
29	K79 II 22 23	KT	playground	11.49	287.09	1337.86
30	K80 II 22 23	KT	playground	30.40	514.09	1176.23
31	K81 II 22 23	KT	playground	1.37	50.69	1631.02
32	K82 II 22 23	KT	playground	2.87	104.30	208.54
33	K83 II 22 23	KT	playground	2.82	158.25	365.82
34	K85 II 22 23	KT	playground	3.40	162.65	440.08
35	K86 II 22 23	KT	playground	5.53	256.12	720.65
36	K96 II 22 23	SW	playground	1.22	50.84	1138.83
37	K97 II 22 23	SW	playground	0.30	7.50	225.29
38	K98 II 22 23	SW	playground	1.37	59.98	66.02
39	K99 II 22 23	SW	playground	2.93	118.70	262.55
40	K100 II 22 23	SW	playground	1.72	72.85	509.96
41	K101 II 22 23	SW	playground	10.20	355.38	477.05
42	K103 II 22 23	SW	playground	5.61	186.89	1220.12
43	K105 II 22 23	SW	playground	13.96	658.42	545.63
44	K106 II 22 23	SW	playground	4.47	139.25	484.54
45	K107 II 22 23	SW	playground	1.14	153.81	214.92
46	K108 II 22 23	SW	playground	0.88	27.70	108.14
47	K109 II 22 23	SW	playground	3.51	123.44	763.28
48	K110 II 22 23	SW	playground	9.71	279.65	2028.63
49	K113 II 22 23	SW	playground	0.23	29.42	43.66
50	K117 II 22 23	PS	playground	20.90	1334.00	3528.37
51	K118 II 22 23	PS	playground	5.40	487.99	1417.86
52	K119 II 22 23	PS	playground	17.04	282.31	1345.92
53	K120 II 22 23	PS	playground	4.85	534.56	1663.11
54	K121 II 22 23	PS	playground	2.24	69.25	519.44
55	K122 II 22 23	PS	playground	1.27	97.80	298.66
56	K123 II 22 23	PS	playground	2.28	160.84	321.18
57	K124 II 22 23	PS	playground	5.60	266.53	773.72

58	K125 II 22 23	PS	playground	0.72	23.98	50.43
59	K126 II 22 23	PS	playground	22.73	940.67	1313.16
60	K127 II 22 23	PS	playground	2.11	73.74	343.03
61	K128 II 22 23	PS	playground	10.19	275.00	1784.55
62	K129 II 22 23	PS	playground	5.38	207.41	651.95
63	K56 II 22 23	KT	field/gym	7.63	419.94	931.49
64	K57 II 22 23	KT	field/gym	3.54	183.20	373.01
65	K64 II 22 23	KT	field/gym	1.07	83.63	173.74
66	K75 II 22 23	KT	field/gym	40.25	952.77	4743.33
67	K76 II 22 23	KT	field/gym	12.71	421.40	1319.00
68	K87 II 22 23	KT	field/gym	7.48	235.18	1107.36
69	K88 II 22 23	KT	field/gym	1.17	64.88	157.89
70	K89 II 22 23	KT	field/gym	1.27	44.82	123.87
71	K90 II 22 23	KT	field/gym	1.06	61.08	137.25
72	K94 II 22 23	SW	field/gym	11.92	407.19	2022.95
73	K95 II 22 23	SW	field/gym	6.36	196.85	1126.73
74	K104 II 22 23	SW	field/gym	8.58	238.88	1645.90
75	K131 II 22 23	PS	field/gym	17.45	561.17	1942.52
76	K132 II 22 23	PS	field/gym	10.21	300.40	1355.22
77	K133 II 22 23	PS	field/gym	2.90	110.29	338.93

**KT – Katowice-Szopienice, SW – Świętochłowice-Lipiny, PS – Piekary Śląskie-Orzeł Biały*

***Other: educational paths, squares, parks, and green areas*
